# Mutation of KIT in cellular extraskeletal myxoid chondrosarcoma: a case report and literature review

**DOI:** 10.1186/s13000-022-01222-7

**Published:** 2022-04-29

**Authors:** Chen Wang, Zhi-Jie You, Xiao-Yan Chen, Jie Lin, Yi-Juan Wu

**Affiliations:** 1 Department of Pathology, Fujian Provincial Hospital, Shengli Clinical Medical College of Fujian Medical University, NO. 134, East Street, Gulou District, 350001 Fuzhou, Fujian Province China; 2 Department of Pathology, Fujian Provincial Hospital South Branch, 350028 Fuzhou, Fujian Province China

**Keywords:** *KIT*, EMC, *NR4A3*, *EWSR1*, Case report

## Abstract

**Background:**

Extraskeletal myxoid chondrosarcomas (EMCs) are solid tumors that have been genetically and biologically characterized. Only a few studies have discussed the role of the *KIT* gene or CD117 expression in EMCs, identified by immunohistochemical (IHC) staining. Herein, we present a novel case of cellular EMC exhibiting an *EWSR1-NR4A3* fusion, *KIT* exon 13 mutations and strong diffuse expression of CD117.

**Case presentation:**

A 69-year-old man presented with a fist-sized tumor on his left shoulder. CT revealed a tumor in the left thoracic and dorsal muscle space. The tumor was completely resected. Histologically, the tumor cells had a nodular structure and infiltrated the peripheral fat and muscle tissues. The tumor cells were uniform in size with round nuclei, well-defined nucleoli and eosinophilic cytoplasm. Immunohistochemically, the tumor cells were positive for CD117, vimentin, CD56 and NSE and focally expressed desmin; the cells were negative for myogenin, S-100, SYN, INSM1, CD34, STAT6, INI-1, Brachyury, ERG, TLE1, AE1/AE3, WT-1, CD99 and SMA. NGS revealed an *EWSR1-NR4A3* fusion and *KIT* exon 13 mutations. The patient had no further treatment after surgery, and no recurrence or metastasis occurred during the ~ 10 month follow-up period.

**Conclusions:**

Molecular detection is an indispensable technique for diagnosing cellular EMCs. The *KIT* mutations noted in this case report may offer fresh insights into EMCs treatment options.

## Background

Extraskeletal myxoid chondrosarcomas (EMCs) are solid tumors that have been genetically and biologically characterized [1], and they comprise < 1% of all soft-tissue sarcomas [2]. Cellular EMCs account for ~ 29% of all EMCs [3] and have the same nodular structure as classic EMCs; however, cellular EMCs demonstrate an abundance of compact tumor cells and a limited myxoid matrix. *EWSR1-NR4A3* fusion products are detected in approximately 62–75% of patients with *NR4A3* rearrangement [4–6]. Only a few studies have discussed the role of the *KIT* gene or CD117 expression in EMCs, identified by immunohistochemical (IHC) staining. Here, we present a novel case of cellular EMC in the left shoulder of a 69-year-old man who exhibited, in addition to the *EWSR1-NR4A3* fusion, *KIT* exon 13 mutations, as revealed by next-generation sequencing (NGS). Moreover, the IHC staining results demonstrated strong diffuse expression of CD117. To the best of our knowledge, this is the first report of *KIT* exon 13 mutations in cellular EMC.

## Case presentation

**Fig. 1:**
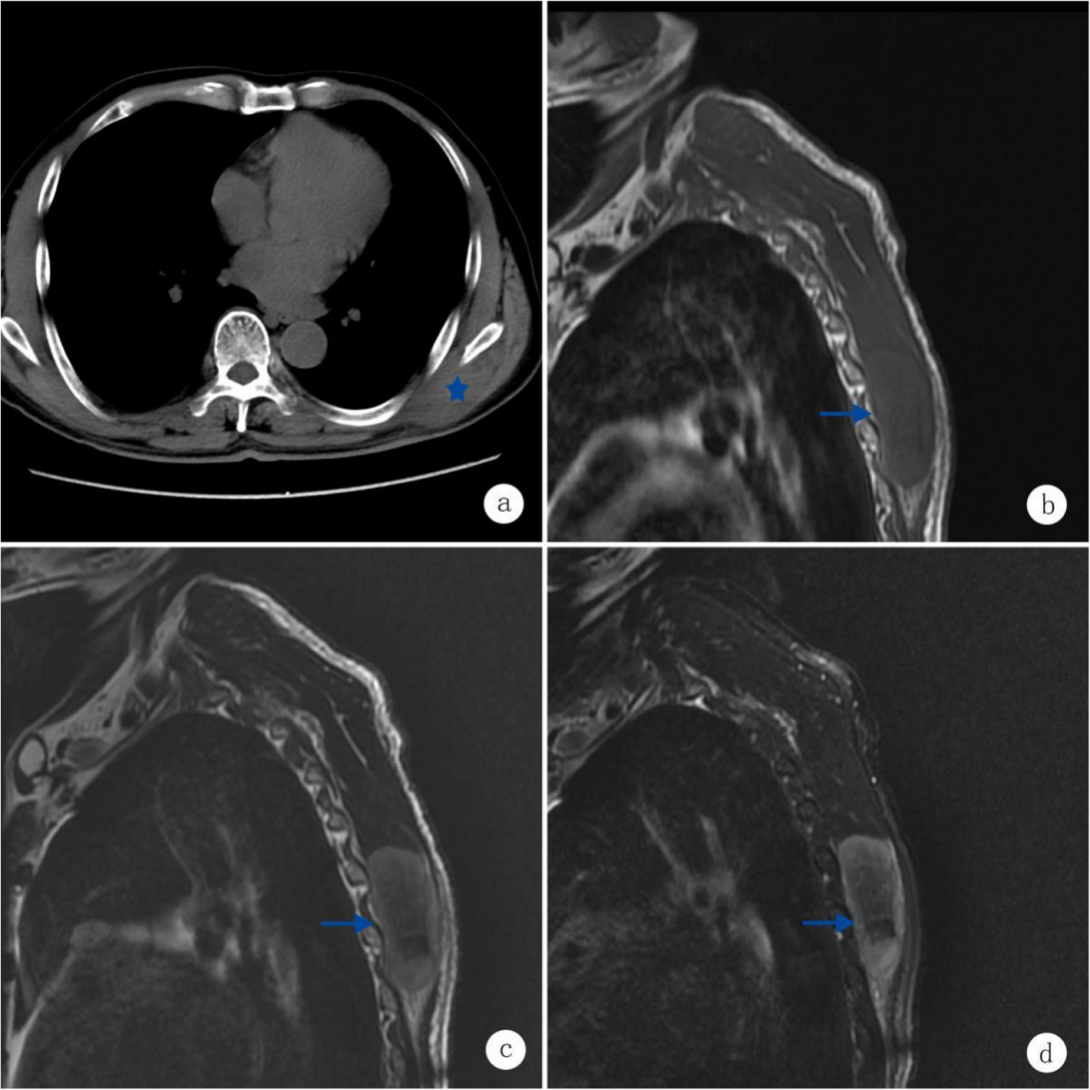
Imaging examination. **a** CT revealed an irregularly shaped soft tissue mass 7.0 cm × 5.4 cm × 2.7 cm in size in the left thoracic and dorsal muscle space, and the mass showed a clear boundary and uniform density. **b** MRI confirmed the presence of an irregularly shaped soft tissue mass in the left thoracic and dorsal muscle space with long T1. **c** T2 signals, as well as a uniform low signal on T1WI. **d** a high signal with fat suppression on T2WI. The blue five-pointed star and arrow indicate the mass

**Fig. 2:**
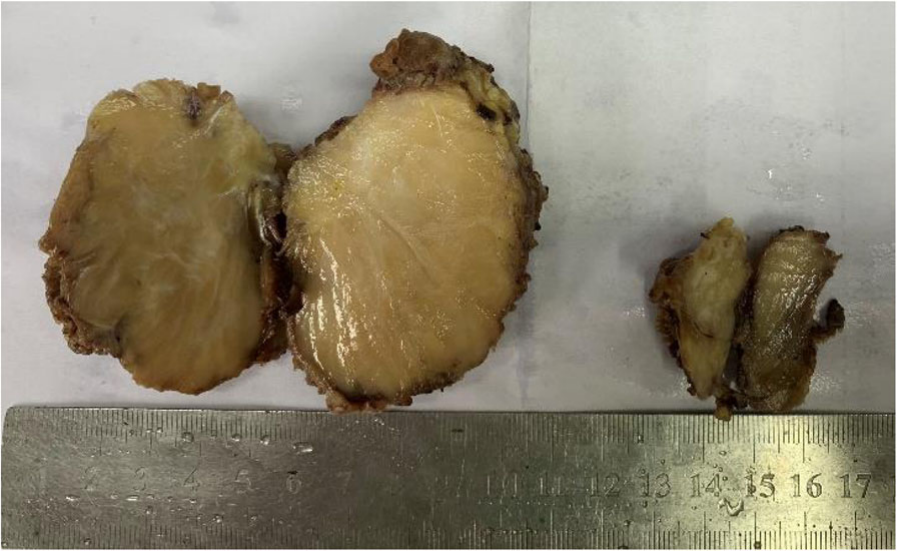
Gross features. The specimen detected was a block of tissues (2 blocks) 7.5 cm × 5.1 cm × 3.3 cm in total size, without a capsule, showing a nodular cutting surface, a grayish-white color and a glossy appearance

Six months before admission, a 69-year-old man noted a fist-sized tumor on his left shoulder without obvious fever, ulcer, night sweating, weight loss or other symptoms. We noted a palpable subcutaneous mass on the left side of the scapula, which was difficult to reach, with a clear boundary, poor mobility and no obvious tenderness. CT revealed an irregularly shaped soft-tissue mass 7.0 × 5.4 × 2.7 cm in size in the left thoracic and dorsal muscle space, and examination of the mass revealed a clear boundary and uniform density (Fig. 1a). The examination of the bone adjacent to the mass revealed slight absorption and destruction. MRI confirmed an irregularly shaped soft-tissue mass in the left thoracic and dorsal muscle space with long T1 and T2 signaling and uniform low-signal intensity on T1WI (Fig. 1b and c) and high-signal intensity with fat suppression on T2WI (Fig. 1d). During surgery, a mass approximately 7 × 6 × 3 cm in size was located in the deep surface of the latissimus dorsi muscle. Most of the mass capsule was complete, some tissues adhered to the bottom near the scapula region, and the mass was removed. The mass had a soft consistency and a pale-yellow appearance. The tissue specimen was then sent to the pathologist for analysis. The specimen comprised a block of tissues (2 blocks), which was 7.5 × 5.1 × 3.3 cm in size, without a capsule and revealed a nodular cut surface, grayish-white in color with a glossy appearance (Fig. 2).

**Fig. 3:**
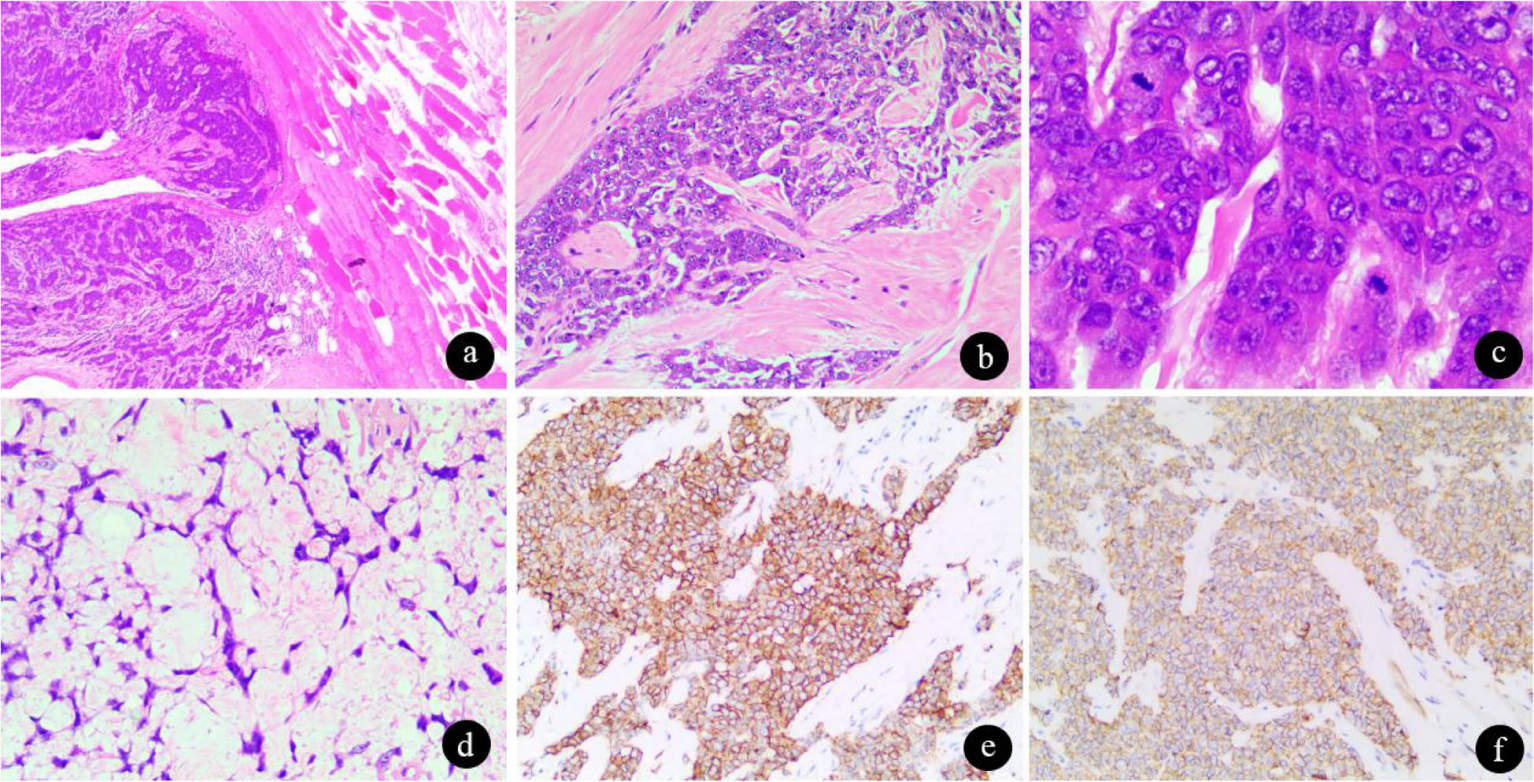
The microscopic and immunohistochemical features. **a** The tumor cells were arranged in a nodular shape and showed infiltrating growth into the surrounding fat and muscle tissue (H&E, × 100). **b** The nodules were separated by a large volume of fibrous tissues (H&E, × 200). **c**: Cells showed uniform size, round nuclei, nonobvious entoblasts, and slightly stained cytoplasm, and mitosis could be seen in highly cellular areas (H&E, × 400). **d** In a few nodules, the cells were loosely arranged and connected into a reticulated or crossed appearance in the myxoid stroma (H&E, × 100). **e** Immunohistochemically, the tumor cells were positive for CD117(× 200). **f** Immunohistochemically, the tumor cells were positive for CD56(× 200)

Microscopically, the tumor cells were arranged in a nodular manner, and infiltrating growth into the surrounding fat and muscle tissue was present (Fig. 3a). The nodules were separated by a large volume of fibrous tissue, and most (> 80%) were solid in texture and comprised round, slightly short spindle cells (Fig. 3b). The cells were uniform in size and had a round nucleus, nonobvious entoblast and slightly stained cytoplasm; mitosis could be seen in high cellularity areas (Fig. 3c). Additionally, in some nodules, the cells were loosely arranged and connected into a reticulated or crossed appearance in the myxoid stroma (Fig. 3d), with no necrosis within the tumor.

IHC staining plays an essential role in this process; thus, the expression of several markers was assessed. The tumor was positive for CD117 (Fig. 3e), Vim, CD56 (Fig. 3f) and NSE and showed focal expression of desmin but was negative for myogenin, S-100, SYN, INSM1, CD34, STAT6, INI-1, Brachyury, ERG, TLE1, AE1/AE3, WT-1, CD99 and SMA.

**Fig. 4:**
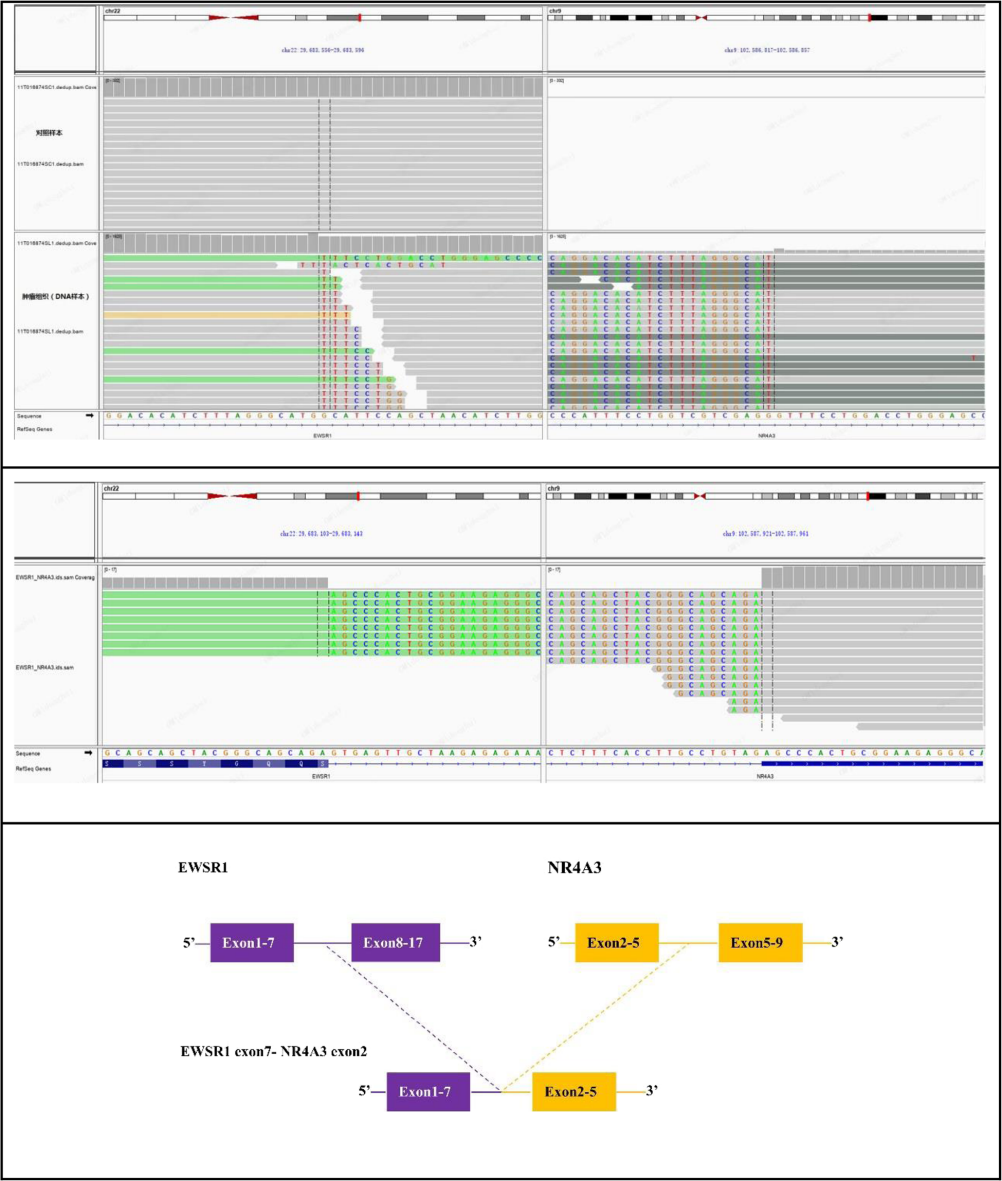
NGS revealed the *EWSR1-NR4A3* fusion was identified by DNA next-generation sequencing (above) and RNA next-generation sequencing (below)

**Fig. 5:**
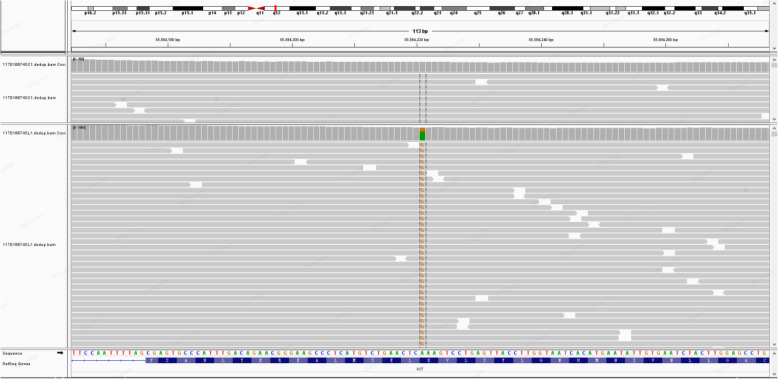
NGS revealed a single nucleotide variant (A to G) in exon 13 of *KIT*

**Fig. 6:**
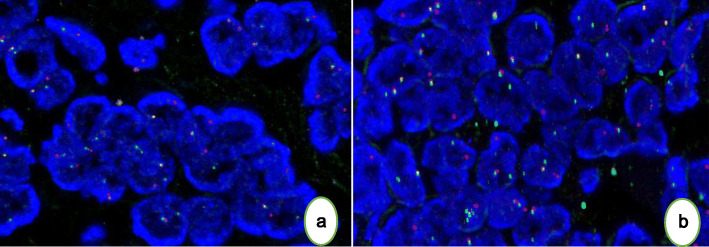
Representative FISH images with *EWSR1* (a) and *NR4A3* (b) showed split red–green staining, indicating the *EWSR1-NR4A3* fusion

Next-generation sequencing (NGS) was performed to detect tumor mutations. NGS detected a translocation of a fragment from exon 1 to 7 of *EWSR1* and exon 2 to 5 of *NR4A3* (Fig. 4a). NGS detected a single-nucleotide variant (A to G) in exon 13 of *KIT* (Fig. 5). Fluorescence in situ hybridization (FISH) confirmed the presence of the *EWSR1-NR4A3* fusion gene. FISH for *EWSR1* and *NR4A3* demonstrated red–green staining, indicating the *EWSR1-NR4A3* fusion (Fig. 6). A diagnosis of cellular EMC was confirmed based on the combined results of the morphological, immunophenotypic and molecular analyses.

## Discussion

EMCs were first defined as a class of solid tumors by Enzinger and Shiraki [1], and they constitute < 1% of all soft-tissue sarcomas [2]. EMCs are commonly seen in male adults, with a mean age of onset of 50 years. Lesions are most frequently found in the deep soft tissues of the proximal extremities, especially the lower extremities; however, in some cases, EMCs can also affect the trunk, head and neck, abdominal wall, paravertebral soft tissues and bones [7–10].

Histologically, EMCs can be categorized into classical EMCs and two variants, cellular EMCs and solid nonmyxoid EMCs [11]. The tumor cells, in this case, had a nodular structure and infiltrated the peripheral fat and muscle tissues. There were abundant fibrous tissues between the nodules, most of which were identified as solid nodules (> 90%) comprised of round or fusiform cells. The tumor cells were uniform in size and had round nuclei with well-defined nucleoli and eosinophilic cytoplasm, and their nuclear fission was 2/10 HPF. The nodular structure partly resembled that of classic EMCs, but the tumor cells were loosely arranged, forming reticular or crosswise layers in the myxoid matrix. Local necrosis was observed. Diagnosing EMC is extremely challenging because of the lack of specificity in their morphological features [12]. Many soft tissue tumors have similar morphology to EMC. The main differential diagnosis includes cellular EMC, proximal-type epithelioid sarcoma, extrarenal malignant rhabdoid tumor, epithelioid angiosarcoma, malignant solitary fibrous tumor, extraosseous Ewing’s sarcoma, desmoplastic small round cell tumor, metastatic dedifferentiated chordoma, poorly differentiated synovial sarcoma and epithelioid malignant peripheral nerve sheath tumor. IHC staining plays an essential role in the diagnostic process. In this case, the IHC staining results suggested that the patient’s tumor was diffusely positive for CD117, vimentin, CD56 and NSE and focally positive for desmin, negative for other markers, and had a ki-67 level of ~ 40%. Despite the effectiveness of IHC staining, a definitive diagnosis of EMC cannot be made solely from positive IHC results for CD117, vimentin, CD56 and NSE as specific markers; however, IHC provided a diagnostic clue, and NGS was performed on the tumor. An EWSR1 exon 7-NR4A3 exon 2 fusion was subsequently identified. Thus, a diagnosis of cellular EMC was confirmed by considering the combined results of the morphological, immunophenotypic and molecular analyses. A recent study [13] reported positivity for INSM1 in up to 90% of EMC cases, which is considered evidence for the neuroendocrine differentiation of EMC cells; however, INSM1 expression was not detected in this case. In terms of molecular genetics, *EWSR1-NR4A3* fusion products are detected in approximately 62–75% of patients with *NR4A3* rearrangement [4–6]. Other studies have reported *HSPA8-NR4A3* translocation [14], a novel t (2;22) (q34; q12) *EWSR1* translocation [15] and *SMARCA2-NR4A3* fusion [16].

Interestingly, along with the *EWSR1* exon 7-*NR4A3* exon 2 fusion, NGS detected *KIT* exon 13 mutations; moreover, it was noted that the IHC staining results demonstrated strong diffuse expression of CD117. Only a few studies have discussed the role of evaluating CD117 positivity and *KIT* gene alterations in EMC diagnostics. Hornick and Fletcher [17] reported that 2 of 20 patients tested positive for CD117, but did not investigate the *KIT* gene. Subramanian et al. [18] conducted IHC and ISH (In Situ Hybridization) tests, revealing that 6 of 11 EMCs were diffusely positive for CD117,*KIT* exons 9, 11, 13 and 17 were screened for mutations in the diffuse positive cases (6/11), and the results suggested indicated no mutations. The IHC staining results in the study by Stacchiotti et al. [19] suggested the presence of CD117 expression in 6 of 9 EMCs cases, however, no *KIT* gene analysis was conducted. Subsequently, Urbini et al. [20] reported the case of an EMC patient (1/20) with *KIT* exon 11 mutations, which, apart from the case presented in this paper, is firstly reported EMC case with *KIT* gene mutations; however, no IHC staining results were conducted. In the present case, the presence of *KIT* exon 13 mutations was established based on NGS and morphological findings, i.e., abundant tumor cells, scarce myxoid stroma and strong diffuse expression of anti-CD117 antibody in tumor cells. We hypothesize that CD117-positive (CD117^+^) cellular EMCs might have a higher frequency of *KIT* gene mutations. EMC subtypes were not elaborated in the abovementioned studies on patients without *KIT* gene mutations who tested positive for anti-CD117 antibodies or those with *KIT* gene mutations identified before anti-CD117 antibody testing. Moreover, to the best of our knowledge, no study has explored the association between anti-CD117 antibodies and the *KIT* gene and EMC subtypes. Therefore, it is necessary to study more EMC cases positive for anti-CD117 antibody expression and cellular EMC cases positive for CD117 expression.

In gastrointestinal stromal tumors, the location of *KIT* mutations is associated with tumor biological behavior, and exon 11 and 13 mutations provide evidence regarding the tumors’ malignant biological status [21]. *KIT* exon 13 mutations are relatively rare, accounting for 0.8–4.1% of all *KIT* mutations, and patients with *KIT* exon 13 mutations benefit from sunitinib therapy [22]. A patient with EMC who had *KIT* exon 11 mutations and had not previously received sunitinib was reported to benefit from sunitinib therapy [20]. However, Brooke et al. [23] reported that stable disease was maintained with imatinib treatement for 3 years in a 55-year-old woman diagnosed with EMCs with *KIT* exon 11 mutations. In the present case, the patient had *KIT* exon 13 mutations but was not administered sunitinib; therefore, whether sunitinib would benefit this patient remains unclear. The 10-year survival rate was 70% among the patients with EMCs, and the prognosis of EMCs was not associated with histological grade or proliferative markers but with the location and size of the tumor in elderly patients [24]. At the time of writing, follow-up on the present case had been conducted for only ~ 10 months; thus, the significance of the follow-up results is limited. We will continue to follow up with this patient.

## Conclusions

Consequently, cellular EMC is a rare tumor type and shows some clinically and biologically unique features. Molecular detection is an indispensable technique for diagnosing cellular EMCs. The *KIT* mutations reported in this case report may offer fresh insights into EMCs treatment options. We firstly reported the case of *KIT* exon 13 mutations, and reviewed the relevant literature to make a deeper understanding of the disease, and provide useful parameters for further gene therapy.

## Data Availability

All data generated or analyzed during this case are included within the article.

## Abbreviations

EMCs: Extraskeletal myxoid chondrosarcomas
IHC: Immunohistochemical
CT: Computed tomography
FISH: Fluorescence in situ hybridization
ISH: In Situ Hybridization
NGS: Next-generation sequencing
MRI: Magnetic resonance imaging
T1WI: T1-weighted image
T2WI: T2-weighted image
